# Dyspnœa on Falling Asleep

**Published:** 1882-07

**Authors:** E. A. Ballard

**Affiliations:** Chicago


### DYSPNŒA ON FALLING ASLEEP.

E. A. Ballard, M. D., Chicago.

In the March issue of The Homœopathic Physician (which, owing to absence from home, has but recently fallen into my hands), is another of Dr. Berridge's very instructive clinical reports, in which the above distressing symptom plays a prominent part.

On page 238 of Lippe's *Repertory*, I find, "Sleep prevented by dyspnœa, *Psor.*, *Ran. bulb.*," and more direct, on page 240, is, "When falling asleep, dyspnœa as if he would suffocate, *Graph.*" "Constriction of chest," is added to this symptom in Hering's Condensed. In latter work, under *Arum tri.*, is, "On falling asleep, feels as if she would smother, starts as if frightened." Of the remedies which Dr. Hale states has relieved this symptom, *Ant. tart.* and *Lach.*, have been verified by myself. In a very critical case of diphtheria, cured with one dose of *Lach.*$^{cm}$, this symptom was present. Some years ago I reported two cases in which *Ant. tart.* had a prompt and curative effect. As clinical evidence, they may be worthy a place in your valuable journal.

The first case was that of my wife. On the third evening, after an easy and natural parturition, I was called suddenly to the bedside. The nurse stated that immediately after going to sleep the breath would become shorter and shorter, and then seem to cease, when the patient would awake, gasping for breath. This had recurred a number of times before I was called. With cessation of breathing, the nurse reported a like cessation of the pulse. The patient said that she experienced a sensation of sinking away while she struggled to retain her breath. *Ant. tart.*$^{200}$, was put on the tongue. She went to sleep soon after, and had no return of the symptom.

The second case was that of a lady about 70 years old. After a long and very severe chill, she was much exhausted and unable to keep awake. No sooner would she close her eyes in sleep than she experienced a sensation of her "breath leaving her body," and she awoke, gasping for breath. After this had recurred a number of times, I was recalled. One dose of *Ant. tart.* had the same effect as in the first case.

The first remedy Dr. Berridge gave his patient was *Syphilinum*, because the symptoms were worse from midday to daybreak. From waning of day to the coming of day I have thought was the time of aggravation under this remedy. At the International Convention, in London, last summer, Dr. R. N. Foster, of this city, reported the cure of a case in which the time of aggravation was from 2 to 5 A. M. After other remedies failed, he gave a dose of *Syphilinum*$^{cm}$, with above result. No doubt the case was one of syphilitic ophthalmia, and the remedy was an exact simillimum. *Syphilinum* is not the only remedy that has aggravation through the night, and should not be given for that symptom alone. On page 292 of Lippe's *Repertory* is, "Pains are aggravated in the evening and do not diminish until daybreak, *Colch.*" According to this same excellent *Repertory*, *Ant. tart.* is indicated where there is aggravation in the afternoon, evening and night. In the *Guiding Symptoms* may be found, "Considerable aggravation toward evening, continuing all night." This indication will be found valuable in other troubles than toothache. Add to this the fact that *Ant. tart.* is one of our most potent remedies wherever and whenever the pneumogastric nerve is involved; that this remedy covers so many of the symptoms of Dr. Berridge's case, especially the most prominent and important one of all, one may be pardoned for asking, after considering all things, if *Ant. tart.* would not have been the best remedy to commence the case with, and if it would not have prevented many symptoms so indicative of this remedy that afterwards appeared?

